# Effect of Plasma-Activated Water on the Cellulase-Producing Strain *Aspergillus niger* A32

**DOI:** 10.3390/jof10080568

**Published:** 2024-08-12

**Authors:** Zhiqing Song, Yingwei Jiang, Chan Chen, Changjiang Ding, Hao Chen

**Affiliations:** 1 College of Electric Power, Inner Mongolia University of Technology, Hohhot 010080, China; ding9713@163.com; 2 Application Laboratory for Discharge Plasma & Functional Materials, Inner Mongolia University of Technology, Hohhot 010051, China; 20211100065@imut.edu.cn (Y.J.); 20211100070@imut.edu.cn (C.C.); chh1126@imut.edu.cn (H.C.); 3 College of Science, Inner Mongolia University of Technology, Hohhot 010051, China

**Keywords:** PAW, CAW, treatment, *A. niger*, mechanism

## Abstract

To investigate the effect and mechanism of plasma-activated water (PAW) on *Aspergillus niger*, PAW was prepared using a needle array–plate dielectric barrier discharge plasma system. The concentrations of long-lived reactive oxygen and nitrogen species (RONS), namely, H_2_O_2_, NO_2_^−^, and NO_3_^−^, in the PAW were 48.76 mg/L, 0.046 mg/L, and 172.36 mg/L, respectively. Chemically activated water (CAW) with the same concentration of long-lived RONS was also prepared for comparison. *A. niger* A32 was treated with PAW and CAW. After treatment, the treated strains were observed and analyzed with scanning electron microscopy (SEM) and transmission electron microscopy (TEM) to screen probable mutants. The results indicated that the pH, conductivity, and ORP values of PAW were 2.42, 1935 μS/cm, and 517.07 mV, respectively. In contrast, the pH and ORP values of CAW were 6.15 and 301.73 mV, respectively, which differed significantly from those of PAW. In addition, the conductivity of CAW showed no change. SEM and TEM analyses revealed that *A. niger* A32 treated with CAW exhibited less damage compared with the control. In contrast, *A. niger* A32 treated with PAW showed significant shrinkage, deformation, and exudate attachment over time. Following PAW treatment, after four passages, a high cellulase-producing stable mutant strain A-WW5 was screened, exhibiting a filter paper enzyme activity of 29.66 U/mL, a cellulose endonuclease activity of 13.79 U/mL, and a β-glucosidase activity of 27.13 U/mL. These values were found to be 33%, 38%, and 2.1% higher than those of the original fungus sample, respectively. In total, 116 SNPs and 61 InDels were present in the genome of the mutant strain A-WW5. The above findings indicate that the impact of PAW on *A. niger* is not only attributed to long-lasting H_2_O_2_, NO_2_^−^, and NO_3_^−^ particles but also to other short-lived active particles; PAW is expected to become a new microbial breeding mutagen.

## 1. Introduction

Discharge plasma, known as the fourth state of matter, encompasses electrons, ions, neutral particles, electric fields, ultraviolet radiation, and other components. As a novel form of comprehensive physical and chemical treatment technology, it offers the benefits of environmental sustainability, straightforward operation, and effective mutagenic impact. Consequently, it has garnered widespread attention from researchers. Nowinski et al. discussed the impact of multiple sublethal doses of non-thermal plasma treatment on the phenotypic changes in fungal cells and the reduction in plant pathogenicity in *Fusarium oxysporum*, *Botrytis cinerea*, and *Alternaria alternata*. Research indicates that subjecting plant pathogenic fungi to multiple treatments with non-thermal plasma not only induces significant morphological alterations in their mycelial structure but also leads to physiological interference. An application of sublethal doses of plasma energy to mycelia can result in decreased pathogenicity in *Fusarium oxysporum*, *Botrytis cinerea*, and *Alternaria alternata* [1]. Wang et al. investigated the bactericidal effect and mechanism of contact glow discharge plasma (CGDP) on *Actinomucor elegans*. They concluded that CGDP has a significant bactericidal effect on *Actinomucor elegans*, primarily through its ability to disrupt the integrity of the cell membrane, thereby achieving an inactivation effect [2]. In order to investigate the efficacy and mechanism of fungal inactivation, Xu et al. utilized cold atmospheric plasma (CAP) for the treatment of yeast in water. Their studies have indicated that short-lived particles such as ·OH and ^1^O_2_ play pivotal roles in yeast inactivation. Furthermore, the synergistic effect of ^1^O_2_ with ROS generated by other plasmas was also unveiled [3]. Lunov et al. primarily discussed the impact of non-thermal plasma on bacteria and fibroblasts and delved into the mechanism behind the plasma sterilization effect and the differentiation between a plasma-triggered effect and ozone (a component of air plasma gas). They substantiated that the safety of air plasma on fibroblasts is a crucial consideration in the application of plasma medicine [4].

As an indirect mode of action, PAW can introduce reactive oxygen and nitrogen species (RONS) into relevant targets within organisms, playing a pivotal role in biological effects. Importantly, this process does not result in secondary pollution during its action on organisms. This aqueous solution, following plasma activation treatment, has distinct advantages in the fields of sterilization, disinfection, and biomedicine due to its uncomplicated preparation, effective treatment results, and environmental friendliness. Xu et al. investigated the inactivation effect of the continuous use of cold atmospheric plasma (CAP) and plasma-activated water (PAW) on two fungi: *Saccharomyces cerevisiae* and *Aspergillus flavus*. Their study demonstrated that the combination of short-term CAP treatment and long-term PAW treatment significantly reduced the abundance of these two fungi compared with a single CAP method while requiring a shorter treatment time. This synergistic effect was attributed to increased membrane damage induced by the short-lived particles ^1^O_2_ and ONOO^−^ in cells, resulting in irreparable oxidative damage to the mitochondria and energy metabolism systems [5]. Yao et al. also examined the immediate bactericidal effect and long-term inhibitory effect of PAW on *A. flavus*, finding that the short-lived particle peroxynitrite (ONOO^−^) in PAW plays a key role in fungal inactivation by destroying their cell wall structures, membrane integrity, redox homeostasis, and mitochondrial function. Additionally, PAW exhibited antibacterial properties and inhibited the synthesis of *Aflatoxin B1 (AFB1)* in *Aspergillus flavus*, demonstrating its potential application in fungal control as well as toxin reduction [6]. Lin et al. used PAW to inactivate *Salmonella enteritidis* on shelled eggs and found that PAW could effectively kill *Salmonella enteritidis* with less damage to the eggshell [7].

*A. niger*, a common species in the Aspergillus genus of fungi, have black–brown clusters; spherical conidia; and black or black–brown and smooth or rough mycelia. *A. niger* is widely distributed, has many application areas, and is an important fermentation industrial strain. *A. niger* is often selected as a cellulase-producing strain in industrial production [8,9].

In this study, we used *A. niger* A32 as the starting strain and prepared CAW in a laboratory with the same concentrations of the long-lived particles H_2_O_2_, NO_2_^−^, and NO_3_^−^ as in the used PAW. The similarities and differences in physical and chemical properties between CAW and PAW were compared. PAW and CAW were used to treat the starting strain, *A. niger* A32. The effects of PAW and CAW on the survival rate, probable mutation rate, and microstructure of *A. niger* were explored and the mechanism of PAW was revealed. Finally, a strain with a high cellulase yield was obtained following Congo red primary screening and fermentation re-screening, then genomic resequencing of stable mutant strains to analyze the molecular mechanism of PAW on *A. niger* A32.

## 2. Materials and Methods

### 2.1. Experimental Device

**Figure 1:**
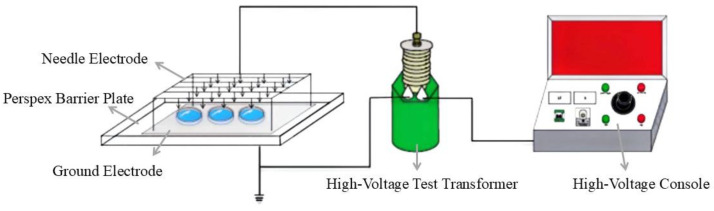
Needle array–plate dielectric barrier discharge device.

A needle array–plate dielectric barrier discharge device was used in this study, as shown in Figure 1. The high-voltage power supply generated an alternating current (AC) and the voltage was continuously adjustable from 0 kV to 50 kV, with a frequency of 50 Hz. The high-voltage electrode consisted of a needle array with a length of 2 cm, a diameter of 1.56 (±0.02) mm, a curvature radius of 0.75 mm, and horizontal and vertical spacing of 4 cm (14 × 7 needles). The ground end was a planar aluminum plate measuring a thickness of 2 mm (85 × 45 cm^2^), which was covered with a plexiglass plate measuring a thickness of 4 mm (100 × 60 cm^2^). The entire electrode system was closed using a 90 × 50 × 15 cm^3^ homemade dark box to create a relatively closed experimental environment for more pronounced plasma effects.

### 2.2. Preparation of Plasma-Activated Water and Determination of H_2_O_2_, NO_2_^−^, and NO_3_^−^ Concentrations

The deionized water was sterilized in a vertical pressure steam sterilizer (model: GI54DS), a measuring cylinder was used to measure 150 mL of sterile water into a polypropylene Petri dish with an inner diameter of 15 cm, and the mass of the sterile water was accurately weighed using an electronic balance with an accuracy of 0.0001 g (model: FD-C3003). The experimental setup used for the study is shown in Figure 1. The high-voltage power supply used for the study had a 50 Hz AC output. The experimental ambient temperature was 22 °C ± 2 °C and the humidity was 30% ± 5%. Referring to the study in [10], the parameters of the plasma-activated water (PAW) were set as follows: the discharge voltage was set to 25 kV, the distance between the needle and media plate was 3 cm, and the activation time of the discharge parameter combination during the preparation of PAW was 180 min.

The concentrations of the long-lived active particles were measured using a visible spectrophotometer (NanoDrop Onec, Thermo Fisher Scientific, Madison, WI, USA) and various kits. A kit for detecting the nitrite content in water and soil (BC1480, Solarbio Technology Co., Ltd. Beijing, China) was applied to detect the NO_2_^−^ concentration in PAW, a kit for determining nitrate ions in water (G0426F Grace Biotechnolgy Co., Ltd. Suzhou, China) was applied to detect the NO_3_^−^ concentration in PAW, and a kit for detecting H_2_O_2_ content (BC3590, Solarbio Technology Co., Ltd. Beijing, China) was applied to detect the H_2_O_2_ concentration in PAW.

### 2.3. Chemically Activated Water Preparation

Based on the measured concentration of long-lived particles in the plasma-activated water, the amounts of NaNO_2_ (in crystalline state), NaNO_3_ (in crystalline state), and H_2_O_2_ liquid were found to be approximately 0.007 mg, 28.85 mg, and 7.31 mL, respectively. These substances were then mixed and dissolved in 150 mL of sterile water to prepare CAW with the same particle concentration.

### 2.4. pH/Conductivity/Redox Potential Determination for PAW and CAW

A portion of the PAW and CAW was extracted and their pH values were measured using a pH meter (model: S210 Mettler-Toledo Shanghai) with an accuracy of 0.01. Their conductivities were determined using a conductivity meter (model: DDS-307A Shanghai Jingke Shanghai) with an accuracy of 0.01 μS/cm. Their oxidation–reduction potentials (ORPs) were measured with a redox potentiometer (model: S210 Mettler-Toledo Shanghai) accurate to 0.01 mV.

### 2.5. PAW and CAW Treatment and Probable Mutant Screening of A. niger A32

#### 2.5.1. Strains

The *A.niger* A32 strain was kindly donated by Associate Professor Aizhi Bai of Inner Mongolia University. The used *A. niger* strain was a wild strain preserved in her laboratory for many years. It was identified by a third-party company as belonging to *A. niger*. The identification report is shown in Supplementary Material Table S1.

#### 2.5.2. Main Reagents and Instruments

Main reagents and drugs: the sodium carboxymethyl cellulose and disodium hydrogen phosphate-citric acid buffer used in this study were produced by Coolaber (Beijing Coolaber Technology Co., Ltd., Beijing, China), while the potassium dihydrogen phosphate, magnesium sulfate, agar powder, glucose, etc., were produced by Xinbote (Tianjin Xinbote Chemical Co., Ltd., Tianjin, China).

#### 2.5.3. Culture Media

The sodium carboxymethyl cellulose (CMC-Na) medium comprised 10 g of sodium carboxymethyl cellulose, 1 g of dipotassium hydrogen phosphate, 0.5 g of magnesium sulfate, 20 g of agar powder, and 1 L of water. The PDA medium comprised 200 g of peeled potatoes; 20 g of dextrose; 20 g of agar powder; and 1 L of water, pH natural. The potatoes were washed, cut into pieces, boiled for 25 min, and filtered twice with 6–8 layers of gauze; glucose, agar powder, and water were added until a volume of 1 L was reached; and then, the medium was sterilized at 121 °C for 30 min. The PDB medium was configured the same way as the PDA medium except that agar powder was not added. All prepared media were stored in a refrigerator at 4 °C.

#### 2.5.4. Preparation of Spore Suspensions of the Strain

The activated *A. niger* A32 strain, preserved in a PDA test tube slant, was inoculated on a PDA plate and cultured at a constant temperature of 28 °C for 5–7 d. When the PDA plate was full of *A. niger*, the spore suspension was transferred to a conical flask with glass beads and shaken at constant temperature for 30 min until the spores were fully dispersed and then, the *A. niger* mycelium was filtered with absorbent cotton. The concentration of *A. niger* spores was calculated using the plate dilution counting method, resulting in a final concentration of *A. niger* spore suspension of 1 × 10^6^ CFU/mL, and then, the sample was stored at 4 °C.

#### 2.5.5. PAW and CAW Treatment of *A. niger* A32

A total of 500 µL of the spore suspension was added to a centrifuge at room temperature and centrifuged for 10 min at 3459× *g*; the supernatant was discarded, while the precipitate was retained. Subsequently, 4.5 mL of pre-prepared PAW and CAW were separately extracted and mixed with *A. niger* A32 precipitation. The treatment process lasted for 30, 60, and 90 min. A sterile water treatment was used as the control group (CK). Following treatment, appropriate gradient dilutions were plated onto CMC-Na medium plates for constant temperature culture at 28 °C for a duration of 7 days. The lethal rate of treatment was calculated using the plate counting method based on the equation in (1), as follows:

(1) $$lethality=\frac{U-T}{U}\times 100\%$$

where *U* is the number of growing colonies without treatment and *T* is the number of growing colonies after treatment.

#### 2.5.6. Screening of High-Yielding Cellulase Strains

Primary sieving: 1 mg/mL Congo red solution (about 30 mL) was added to the CMC-Na plate as described above and stained for 30 min. Then, 1 mol/L NaCl (about 30 mL) was added and soaked for 30 min and any Congo red that was not firmly bound to cellulose was washed off. The diameter of both the transparent ring (D) and the colony (C) [11] were measured using a ruler and the ratio of the transparent ring diameter to the colony diameter (HC) was calculated. Through numerous experiments, we determined that, when the HC value of the strain equaled or exceeded 3.33, it could be identified as a probable mutant and the probable mutation rate was calculated according to Equation (2), as follows:

(2) $$probable\;mutation\;rate=\frac{M}{N+M}\times 100\%$$

where *M* is the number of colonies with an HC value ≥ 3.33 and *N* is the number of colonies with an HC value < 3.33.

Re-screening: The strains with a strong ability to decompose cellulose obtained from the initial screening were cultured at 28 °C on a 180 r/min shaking bed for 6 d. Samples were taken every 24 h and centrifuged in a centrifuge at 3903× *g* for 10 min and the supernatant (enzyme solution) was taken to determine the enzyme activity in order to select the high cellulase-producing strains.

A schematic diagram of the screening steps is provided in the Supplementary Materials Figure S1.

#### 2.5.7. Scanning Electron Microscopy (SEM) Measurements

The fungal suspension was treated and then centrifuged at a low speed to precipitate the cells. The supernatant was subsequently removed and the precipitated cells were washed with 0.1 M sodium phosphate buffer (PBS) to maintain their integrity. Following this, the cells were fixed with 2.5% glutaraldehyde at 4 °C to preserve their original morphology.

To prevent artifacts during observation, dehydration was carried out on a gradient of 50%, 70%, and 100% ethanol to gradually replace moisture in the sample. Additionally, supercritical drying technology was employed to ensure complete removal of ethanol while maintaining the cell structure.

The sample was fixed on conductive tape and sputtering gold palladium increased its conductivity. Detailed observations and photographs of the samples were obtained in the scanning electron microscope’s high vacuum mode in order to acquire clear and stable images.

Finally, scanning electron microscopy was used to observe the effects of PAW and CAW on the surface microstructure of *A. niger* mycelia and spores.

#### 2.5.8. Transmission Electron Microscopy (TEM) Measurements

The pre-treatment steps for TEM determination were conducted in the same manner as those for SEM determination. In contrast to the SEM treatment, a pure acetone solution was utilized to dehydrate the samples in order to better preserve the microstructure and details of the *A. niger* cells. Subsequently, the samples underwent infiltration, embedding, slicing, and staining processes. The impact of PAW and CAW on the ultra-microstructure of *A. niger* was then observed using transmission electron microscopy.

#### 2.5.9. Plotting of Glucose Standard Curves

Glucose standard curve plotting: 0, 0.2, 0.4, 0.6, 0.8, 1.0, 1.2, 1.4, 1.6, and 1.8 mL of a 1 mg/mL glucose standard solution were transferred to clean 50 mL centrifugal tubes and deionized water was added to each tube until a total volume of 2 mL was reached to obtain a series of glucose standard solutions with different concentrations. Then, 1.5 mL of DNS (3,5-dinitrosalicylic acid) reagent was added to each centrifuge tube, the solution was quickly mixed and cooled to room temperature with running water after a boiling water bath for 5 min, and the total volume of the solution was adjusted to 25 mL by adding distilled water to each centrifuge tube. The absorbance of the solution in each tube was measured using a visible spectrophotometer at a wavelength of 540 nm (OD_540_ nm), the total volume of the solution was adjusted to 25 mL based on the measured absorbance, and a standard curve was plotted based on the measured absorbance values with glucose content as the horizontal coordinate (unit: mg/mL) and the corresponding absorbance value (OD_540_ nm value) as the vertical coordinate.

The glucose standard curve was plotted according to the experimental results and the curve equation was y (OD_540_ nm value) = 1.0462 × (glucose content) + 0.0288, as shown in Figure S2, with the correlation coefficient R^2^ = 0.9953, which was linear, and subsequent experiments measuring the OD_540_ nm value were used to determine the glucose content in the samples and thus to calculate the enzyme activity.

#### 2.5.10. Determination of Cellulase Activity

The filter paper enzyme activity (FPA) assay can assess the ability of cellulase to degrade cellulose. Using filter paper as the reaction substrate, qualitative filter paper was cut into strips (1 cm × 6 cm) and put at the bottom of a test tube; 0.50 mL of enzyme solution and 1.50 mL of 50 mmol/L disodium phosphate-citric acid buffer solution at pH 4.6 were added; the solution was held for 60 min at 50 °C in a water bath, then cooled down, put into a boiling water bath for 5 min, and then cool it down; 3 mL of DNS solution was added; and then, the absorbance value at the wavelength of 540 nm was determined using a spectrophotometer. The absorbance value was measured at a wavelength of 540 nm, the glucose content was calculated according to the standard curve equation, and the filter paper enzyme activity was calculated. A blank control was made with distilled water instead of the enzyme solution.

Determination of cellulose endonuclease activity (CMC): With CMC-Na as the substrate, 1 g of CMC-Na was weighed and boiled in distilled water until completely dissolved, with 50 mmol/L, pH 5 disodium phosphate-citric acid buffer, until 100 mL was reached to obtain the CMC-Na solution; then, 0.50 mL of the enzyme solution was added to 1.5 mL of the CMC-Na solution and left in a 50 °C bath for 30 min; after cooling, 2 mL of a 10% sodium hydroxide solution was added and left in a boiling water bath for 5 min; after cooling again, 3 mL of a DNS solution was added and left in a boiling water bath with a spectrophotometer; and then, 3 mL of DNS solution was added again and kept at 50 °C for 30 min. After cooling, 2 mL of 10% sodium hydroxide solution was added, put into a boiling water bath for 5 min, then cooled down again, and 3 mL of DNS solution was added once more. The absorbance value was measured at a wavelength of 540 nm with a spectrophotometer, the glucose content was calculated according to the equation of the standard curve, and the enzyme activity of CMC was calculated. A blank control was made with distilled water instead of the enzyme solution.

Determination of β-glucosidase activity: Salicin was utilized as the substrate. A 1.0 mL aliquot of a 1 g/l solution of salicin (prepared by dissolving 1 g salicin in 100 mL buffer with pH = 5) was mixed with 1.0 mL of enzyme solution and allowed to react at 50 °C for 30 min. Subsequently, 3 mL of DNS reagent was added, followed by boiling in a water bath for 5 min. After cooling, the absorbance value was measured at a wavelength of 540 nm using a spectrophotometer. The glucose content was calculated using the standard curve equation and the β-glucosidase activity was determined accordingly. A blank control using distilled water instead of an enzyme solution was also included in the experiment.

Enzymatic activity definition: Under the specified conditions, the amount of enzyme required to hydrolyze 1 mL of enzyme solution to produce 1 μmol of glucose per minute is one unit of enzyme activity (U/mL).

### 2.6. Gene Resequencing Analysis of Mutant and Wild Strains

Through the DNBSEQ platform, genomic DNA was extracted and randomly interrupted and DNA fragments of the required length were recovered via electrophoresis.

The concentration of DNA samples was detected using a Qubit fluorescence quantitative analyzer and the integrity was evaluated via 1% agarose gel electrophoresis. The qualified samples were broken with a Covaris ultrasonic instrument and 300–400 bp fragments were selected using an Agencourt AMPure XP-Medium kit (Beckman Coulter Trading (China) Co., Ltd., Shanghai, China). After purification, the amount of DNA was detected using a Qubit dsDNA HS Assay Kit. Next, the DNA end was repaired, a base was added, the adapter was connected, and PCR amplification was performed. The amplified products were screened using an Agencourt AMPure XP-Medium and detected with Agilent 2100 Bioanalyze (Agilent Technologies Co., Ltd., Santa Clara, CA, USA). The PCR product was denatured into a single chain, and the single-chain cyclization product was obtained after a cyclization reaction, which was the library. After the library was detected using Agilent 2100 Bioanalyze, DNA nanospheres (DNBs) were formed via rolling circle replication and loaded into the sequencing chip using high-density nanochip technology. Finally, c PAS technology was used for sequencing.

### 2.7. Statistical Analysis

The experimental data were obtained based on three independent measurements and expressed as mean ± standard deviation. Plots were created using Excel 2020 software. Origin 2021 software was used for mapping, and SPSS 18.0 software was used for the significance analysis. A one-way analysis of variance (ANOVA) was used to analyze the differences between the treatment groups and the differences between the groups were determined using a post hoc test. The least significant difference (LSD) and Duncan’s test were significant at *p* < 0.05.

In this study, we measured samples from two different treatment groups to assess their effects. Three independent samples were collected for each treatment group and three repeated measurements were taken for each sample to calculate the mean and standard deviation. SPSS software was used to analyze the data. We calculated the mean value, standard deviation, and confidence interval to describe the characteristics of the sample. We performed a one-way analysis of variance (ANOVA) using SPSS software to assess differences between the treatment groups, followed by multiple comparisons to identify specific significant differences. The statistical significance level was set at *p* = 0.05.

The enzyme activity of *A. niger* A32 from two different treatment groups was analyzed. Each treatment group consisted of seven independent samples, each of which was measured three times to calculate the mean and standard deviation. To assess the differences in enzyme activity between the treatment groups, we used a single-factor ANOVA analysis, followed by multiple comparisons. The statistical significance level was set at *p* = 0.05.

## 3. Results and Discussion

### 3.1. Concentration of Long-Lived Particles in Plasma-Activated Water

H_2_O_2_ is a reactive oxygen species (ROS) in plasma-activated water (PAW) that plays a crucial role in the antimicrobial properties of PAW and contributes to the increased mortality of *A. niger*. As indicated in Table 1, the concentration of H_2_O_2_ in PAW reached 48.76 mg/L, while the concentrations of NO_2_^−^ and NO_3_^−^ were 0.046 mg/L and 172.36 mg/L, respectively. Both NO_2_^−^ and NO_3_^−^ are reactive nitrogen species (RNS) with lethal or inhibitory effects on microorganisms.

In a previous study, Li et al. utilized a needle array–plate dielectric barrier discharge device to prepare PAW for detecting its active components. That study revealed that discharge plasma treatment leads to certain concentrations of H_2_O_2_ and NO_2_^−^. A redox reaction occurred, generating NO_3_^−^. and H_2_O through the reduction in H_2_O_2_ and the oxidation of NO_2_^−^. This chemical reaction is driven by the reducibility of H_2_O_2_, enabling it to oxidize NO2^−^ to form NO_3_^−^ while being reduced to water.

It is worth noting that long-term plasma activation treatment results in a decrease in NO_2_^−^ concentration due to some conversion into NO_3_^−^. This finding holds significant implications for understanding the conversion of active substances and changes in nitrite concentration during plasma-activated water treatment [10].

### 3.2. pH, Conductivity, and Redox Potential of PAW and CAW 

Sterile water was used for the experimental preparation of PAW with an initial pH of 6.39, a conductivity of 7.15 µS/cm, and a redox potential of 407.27 mV. After activation of the sterile water by the needle–array–dielectric blocking discharge device, the PAW showed lower pH, higher conductivity, and higher redox potential compared with the untreated sterile water and the changes in these physicochemical properties were, to some extent, related to the reactive oxygen and nitrogen species (RONS) produced in the action of the discharge plasma with sterile water.

pH is a measurement of the concentration of hydrogen ions in sterile water. Its decrease is due to the formation of acidic chemical species. As shown in Table 2, the pH of the prepared PAW was tested to be 2.42 and the pH of the CAW prepared based on the concentration of the three long-lived RONS in the PAW was 6.15. Conductivity represents the ability of any solution to allow an electric current to pass through it and conductivity increases significantly due to plasma activation, primarily due to the production of ions. The conductivity of the PAW was 1935 µS/cm; however, the formulated conductivity of the CAW was unreadable as the value kept changing during the assay. Oxidation–reduction potential (ORP) can be defined as the ability of any solution to gain or release electrons to the electrode, which is considered an important factor affecting the inactivation of microorganisms and disrupting their cell membranes and defense mechanisms [12]. The higher the ORP value, the higher the oxidizing capacity and the higher the antimicrobial potential. Compared with untreated activated sterile water, PAW had a higher ORP value of 517.07 mV while CAW had an ORP of 301.73 mV.

From the above results, compared with PAW, the pH of CAW was found to be higher, while its ORP was significantly lower. In particular, the ORP of CAW was even smaller than that of CK (sterile water); in addition, the change in the conductivity of CAW lacks obvious regularity. These results suggest that other active particle components are actually present in PAW compared with CAW.

### 3.3. Lethality of A. niger A32

**Figure 2:**
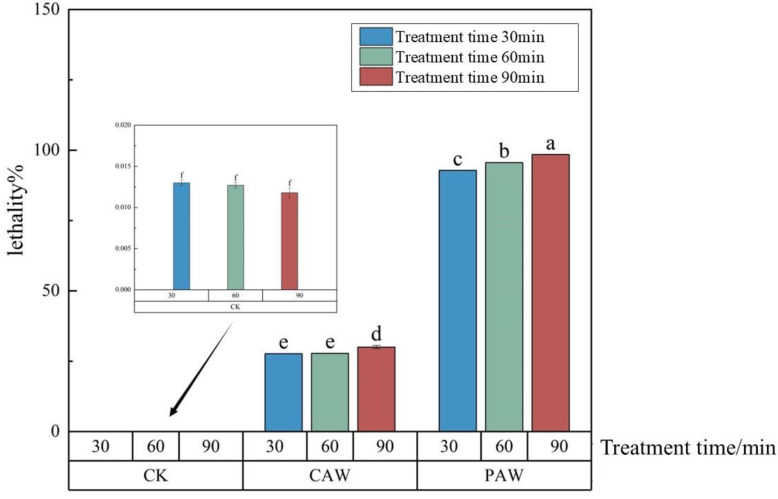
The lethality of *A. niger* was mitigated using plasma-activated water (PAW) and chemically activated water (CAW) with the same long-life concentration of reactive oxygen and nitrogen species (RONS), under the following conditions: a discharge voltage of 25 kV, a needle–dielectric plate distance of 3 cm, and an activation time of 180 min. Different letters indicate significant differences between the sample means (*p* < 0.05).

Comparing the effects of PAW and CAW on *A. niger* A32, from Figure 2, we can see that the lethality of the departing strain, *A. niger* A32, had a significant dose effect with the treatment time of PAW. The lethality of *A. niger* A32 increased with an increase in treatment time. When the treatment time was 90 min, the lethality of PAW was the largest, up to 98.5%, which was much higher than the half lethality. As for the lethal effect of CAW on *A. niger*, no matter how the treatment time changed, its lethality was much higher than that of the CK group but the lethality values of different treatment times did not change much; the lethality of CAW was the largest when the treatment time was 90 min, which was 30.3%, much lower than the semi-lethal rate. This indicates that the treatment impact of PAW on *A. niger* is attributed not only to long-lasting particles such as H_2_O_2_, NO_2_^−^, and NO_3_^−^ but also to other short-lived RONS. Xu et al. discovered that low-temperature plasma can efficiently deactivate harmful microorganisms, including fungi, by generating various reactive oxygen species (ROS) such as hydroxyl radicals (·OH), singlet oxygen (^1^O_2_), hydrogen peroxide (H_2_O_2_), and superoxide anion (O_2_^−^) [5]. Huang et al. discovered that the short-lived species in PAW were more closely associated with the bactericidal effect, while NO, ^1^O_2_, and ONOO^−^ played more significant roles in bacterial inactivation [13].

### 3.4. Microstructural Changes in A. niger

**Figure 3:**
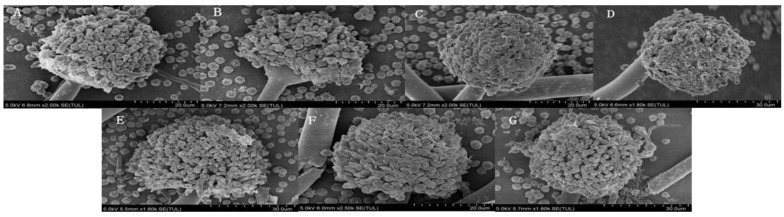
The microstructural changes in plasma-activated water (PAW) and chemically activated water (CAW) with the same long-life RONS concentration were observed under the conditions of a discharge voltage of 25 kV, a needle–dielectric plate distance of 3 cm, and an activation time of 180 min using a scanning electron microscope (SEM). Sample (**A**) is the control group. Samples (**B**–**D**) were treated with PAW for 30 min, 60 min, and 90 min, respectively. Similarly, samples (**E**–**G**) were treated with CAW for 30 min, 60 min, and 90 min.

As can be seen from the scanning electron microscope (SEM) pictures in Figure 3, the microstructure of *A. niger* A32 changed significantly after treatment with PAW compared with that of the control group (CK). The spores of the CK group presented an intact surface and a smooth and full morphology and the conidia of *A. niger* A32 were affected to a certain extent by the increase in treatment time with PAW, as seen in Figure 3B–D, The growth of conidia was not vigorous and the spore surface did not become smooth, wrinkled, or deformed; it had no obvious attachment of lysates, which might be due to the fact that PAW damaged the cell wall of *A. niger* A32 and affected the permeability of its cell membrane during the treatment process, leading to leakage of the internal material of spores [14,15,16,17]. PAW contains a large number of reactive particles, which penetrated into the cell, damaged the normal cellular physiological activities, and disrupted the integrity of *A. niger* A32. Additionally, from Figure 3E–G, we can see that the microstructure of *A. niger* spores after treatment was not significantly different from that of the CK group, regardless of the change in CAW treatment time.

**Figure 4:**
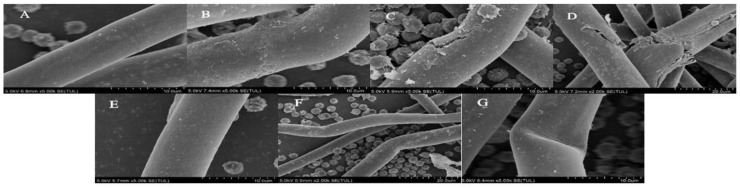
Plasma-activated water (PAW) and chemically activated water (CAW) with the same long-life RONS concentrations were treated under the conditions of a discharge voltage of 25 kV, a needle–dielectric plate distance of 3 cm, and an activation time of 180 min. SEM images of the microstructure change in *A. niger* treated with PAW and CAW with the same long-life RONS concentrations: (**A**) the control group; (**B**–**D**) treatment with PAW for 30 min, 60 min, and 90 min, respectively; and (**E**–**G**) treatment with CAW for 30 min, 60 min, and 90 min, respectively.

As can be seen from the SEM images in Figure 4, the microstructure of the mycelium of *A. niger* A32 after PAW treatment changed significantly compared with that of the control group (CK). The mycelium in the CK group was structurally intact and presented a flat and smooth surface. With the increase in PAW treatment time, as shown in Figure 4B–D, the surface morphology of *A. niger* A32’s mycelium was affected to some extent and the surface morphology of the mycelium underwent significant changes. The original smooth surface became uneven and rough and holes and protrusions even appeared. This change in microstructure is due to the interaction between the active particles in the PAW and the surface of the mycelium, resulting in changes in surface composition and structure. With the increase in treatment time, the surface damage to mycelium was more serious [1,18]. Additionally, from Figure 4E–G, we can see that the microstructure of *A. niger* mycelium after treatment was not significantly different from that of the CK group, regardless of the changes in CAW treatment time.

**Figure 5:**
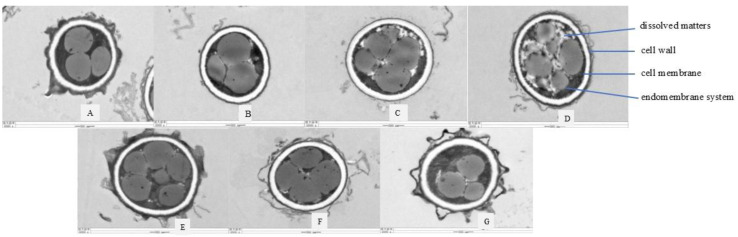
The transmission electron microscopy (TEM) images of *A*. *niger* treated with plasma-activated water (PAW) and chemically activated water (CAW) with the same long-life RONS concentrations under the conditions of a discharge voltage of 25 kV, a needle–dielectric plate distance of 3 cm, and an activation time of 180 min. (**A**) is the control group; (**B**–**D**) were treated with PAW for 30 min, 60 min, and 90 min, respectively; (**E**–**G**) were treated with CAW for 30 min, 60 min, and 90 min, respectively.

From the TEM pictures in Figure 5B–D, we can see that the degree of damage to the inner membrane of *A. niger* A32 after PAW treatment was more and more serious with increasing treatment time; obvious lysates could be seen. Compared with the control group (CK), the change was obvious and the inner membrane of *A.niger* A32 in the CK group was relatively complete. In Figure 5E–G, the inner membrane system was also slightly damaged after CAW treatment and only a small amount of dissolution could be seen. Compared with the PAW group, the damage to the inner membrane system of *A.niger* A32 was lighter but more obvious than that of the CK group.

From the above results, we can see that CAW basically has no effect on the surface microstructure of *A. niger*, so CAW can be presumed to have little effect on the lethality of *A. niger* and the probable mutation rate to be even lower, which basically agrees with the specific results of the lethality and the probable mutation rate obtained earlier. Once again, this shows that the effect of PAW on *A. niger* is caused not only by the long-lived particles H_2_O_2_, NO_2_^−^, and NO_3_^−^ but also by the fact that other short-lived RONS play a great role too. Since PAW caused much more severe damage to *A. niger* than CAW, the probable mutation rate of PAW was higher relative to CAW.

### 3.5. Screening of A. niger A32 Probable Mutant Strains

Table 3 shows the probable mutation rate of *A. niger* A32 treated with PAW and CAW under different treatment times. From the table, we can see that the probable mutation rate of *A. niger* A32 was the highest, at 33.33%, after 90 min of treatment in PAW. The probable mutation rate of *A. niger* A32 was also the highest, at 0.33%, after 90 min of treatment in CAW, and the probable mutation rate was zero for treatment times 30 min and 60 min. Comparing the results for the probable mutation rate of *A. niger* induced by PAW and CAW, we can see that the effect of PAW on *A. niger* is obvious.

From the lethality and probable mutation rate of PAW and CAW on *A. niger* A32, we can see that, although CAW and PAW have the same concentration of the long-lived particles H_2_O_2_, NO_2_^−^, and NO_3_^−^, the values of lethality and probable mutation rate are very much different; the lethality of CAW is only 0.3 times that of PAW; and the probable mutation rate of CAW is even lower, which is only 0.01 times that of PAW. This result suggests that the main factors for the effects of PAW on organisms are not only long-lived particles such as H_2_O_2_, NO_2_^−^, and NO_3_^−^ but also its superoxide anions (O_2_^−^), hydroxyl radicals (·OH), single-linear oxygen (1O_2_), peroxynitrites (ONOO^−^), and hydronium hydrates (H_3_O^+^), which affect the production of biological effects [19,20,21,22,23].

**Figure 6:**
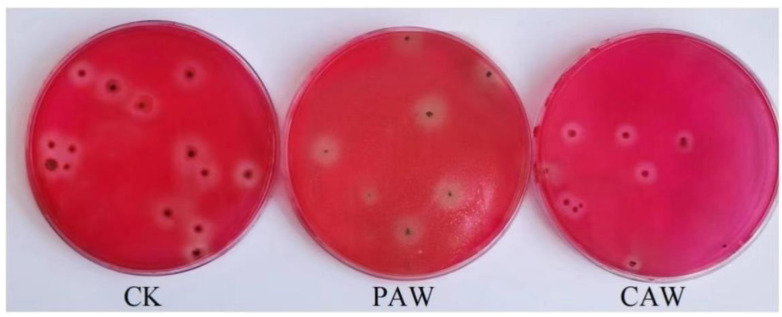
Colony morphology of *A. niger* treated with PAW/CAW.

Figure 6 shows the colony morphology of *A. niger* treated with plasma-activated water and chemically activated water; we can see that the colonies of *A. niger* treated with PAW treatment appeared to be polymorphic, with differences in shape and size.

The seven probable mutant strains obtained from the initial screening after CAW treatment were named C-WW1-C-WW7 and those for the seven probable mutant strains obtained from the initial screening after PAW treatment were named A-WW1-A-WW7. These 14 strains with strong cellulose decomposition abilities were re-screened. During the fermentation re-screening, the enzyme activity of all strains in the PAW group showed tendencies to first increase and then decrease and the enzyme activity of the probable mutant strains reached the highest at 3 d of fermentation.

The HC values and enzyme activity measurements of the probable mutant strains are shown in Table 4 and Table 5. The HC values of the seven probable mutant strains in the PAW primary screening were a little bit larger than the HC screening value of 3.33, whereas the HC values of the CAW group did not differ much from the original strains numerically, which also indicated that the enzyme-producing activity of the probable mutant strains of the primary screening of *A. niger* A32 treated with CAW was smaller than that of the probable mutant strains in the screening of the PAW. Table 4 shows the HC values of *A. niger* A32 treated using PAW, with the results of each enzyme activity determination. Through a comprehensive comparison of the filter paper enzyme activity, cellulose endonuclease enzyme activity, and β-glucosidase enzyme activity, A-WW5 was selected as a high cellulase-producing strain and the activities of the filter paper enzyme, cellulose endonuclease, and β-glucosidase of the probable mutant strain A-WW5 were 29.66, 13.79, 27.13 U/mL, respectively, which were 33%, 38%, and 2.1% higher compared with the departure strain (CK group). Table 5 shows the results of the HC values and the determination of each enzyme activity of *A. niger* A32 treated using CAW and the values of each enzyme activity in CAW-treated *A. niger* A32 were found to not be as good as those of the original strain in the enzyme activity determination. This also further indicates that PAW has other RONS particle effects on the treatment process of *A. niger* A32. For example, the role of PAW in the removal of an *Escherichia coli* (*E. coli*) biofilm was explored by Xia et al. A superoxide anion (O_2_^−^) was found to play a key role in the inactivation of E. coli biofilm with PAW [19]; Gou et al. found that the reactive species produced via cold atmospheric pressure plasma mixed with PAW, especially single-linear oxygen, can effectively inactivate different types of phages in water, including double-stranded DNA, single-stranded DNA, and RNA phages, by destroying nucleic acids and proteins and causing phage aggregation [24]. By studying the effectiveness of plasma-activated chemical solutions (PACSs) in sterilization, Li et al. concluded that PACSs containing H^+^, NO_2_^−^, and H_2_O_2_ were the most effective in killing Escherichia coli and that peroxynitrite (ONOO^−^) was the key to the sterilization of PACS [25]. Liu et al. investigated the effect of PAW on Penicillium (*Penicillium*) by preparing PAW via microhollow cathode discharge (MHCD). The results of their study revealed that HNO_2_ and H_2_O_2_ reactions are important in the inactivation of Penicillium, most likely due to the effect of its product peroxynitrite (ONOOH). The effective effect of ONOOH on inactivation beyond its theoretical lifespan is indirectly reflected in the treatment efficiency and the complexity of the chemistry of peroxynitrite may be responsible for prolonging this effect [26]. Du et al. compared the RONS morphology and antimicrobial effect of He and air SMD plasma by comparing and contrasting the RONS morphology and antimicrobial effect of He and air SMD plasma and found that air SMD plasma exhibited a stronger antimicrobial activity compared with He plasma and that the key antimicrobials for He and air SMD plasma were –OH and O_3_, respectively [27]. Wang et al. investigated the effects of cold atmospheric plasma (CAP) on fungal growth, deoxynivalenol (DON) biosynthesis, and *Fusarium* pathogenicity and found that 1O_2_ diffuses more readily into the cells and leads to lipid peroxidation due to its relatively long lifetime (~2 μs) compared with –OH (~10^−9^ s) and that 1O_2_ is also an important antimicrobial agent in this plasma–liquid system [28]; Cheng et al. used an S-Logistic model to characterize the relationship between the dose of a plasma-activated medium (PAM) and the corresponding cell lethality effect, during which H_3_O^+^ was found to not only affect the oxidation potentials of NO_2_^−^ and NO_3_^−^ but also synergistically act with other species to produce new RONSs. Furthermore, its concentration (pH) directly affects cellular viability [24].

The reactive oxygen species and nitrogen particles (RONSs) produced by the plasma play key roles in the mutagenesis mechanism. These active particles affect the genetic material of the organism in a variety of ways, leading to mutations. Li et al. treated *Cladosporium* B03 using ultraviolet atmospheric-pressure room-temperature plasma compound mutagenesis. The enzyme production ability of the strain was found to significantly improve with ultraviolet mutagenesis and ARTP mutagenesis. The plasma could destroy the phosphodiester bond of nucleotides, enable the cells to initiate their SOS repair mechanisms, and produce a strong distortion effect on the genetic material of microorganisms, thereby inducing the strains to produce genetically stable mutations [29]. Some studies have used atmospheric-pressure room-temperature plasma (ARTP) mutagenesis to increase the yield of astaxanthin in yeast [30,31]. Some researchers have used plasma technology to mutate different yeast strains and obtained corresponding stable mutant strains [32,33,34,35,36,37]. Xu et al. discussed the inactivation effect of atmospheric-pressure low-temperature plasma on Staphylococcus aureus and its possible mechanisms. The experimental results show that plasma treatment may lead to the formation of ROS in bacteria, induce an increase in oxidation pressure in bacteria, and cause bacterial inactivation when exceeding the threshold of bacterial oxidative stress reaction. In addition, plasma treatment may affect the expression of genes related to oxidative stress response, biofilm formation, antibiotic resistance, and DNA damage protection/repair in bacteria [38].

These studies show that the short-lived particles in PAW have significant effects in microbial killing and further indicate that the mutagenic process of *A. niger* A32 is closely related to other RONS particles in PAW.

### 3.6. Genome Resequencing Results and Analysis of Mutant Strains

In order to make the experimental results more accurate and reliable, de novo sequencing analysis was performed on *A. niger* A32 and gene detection was performed on the genome of the strain to determine the gene function and related description information, making it a reference gene sequence. The difference between the mutant strain A-WW5 and the reference strain *A. niger* A32 was thus found.

**Figure 7:**
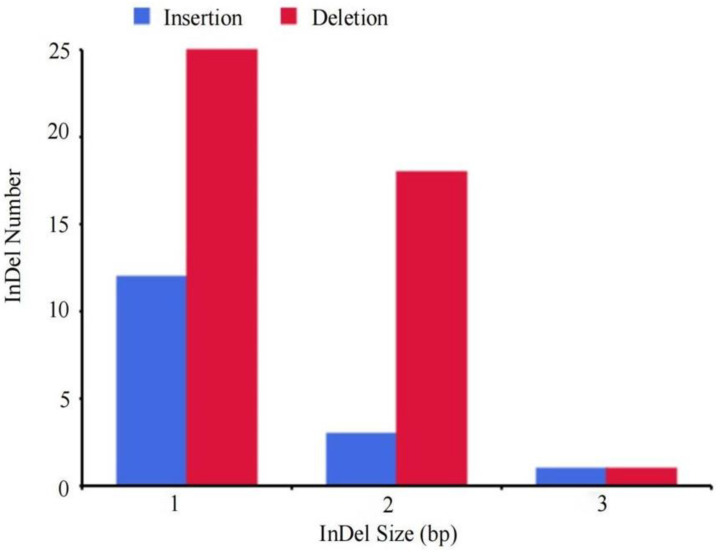
InDel length distribution map.

Statistics for the presence of single-nucleotide polymorphisms (SNPs) in mutant strain A-WW5 are shown in Table 6. Compared with the reference sequence, 116 SNPs were present in the genome of the mutant strain A-WW5 and 69 SNPs in the CDS region. Among them were 56 non-synonymous mutations, accounting for 48.3% of the total, and 13 synonymous mutations, accounting for 11.2% of the total. As shown in Table 7, there are 35 base transversions and 34 base transitions. Among the 47 SNPs in the non-coding region (Intergenic), 31 were base transversions and 16 were base transitions. As shown in Table 6 and Table 7, of the total 116 SNPs detected, 66 base transversions and 50 base transitions occurred. Compared with the reference genome, there were 16 base pair insertions, 44 base pair deletions, and 1 CDS_InDel in the genome of the A-WW5 mutant strain, as shown in Figure 7. Among them, the deletion of base pairs accounted for 72% of the total.

In this study, the probability of non-synonymous mutations was higher than that of synonymous mutations, indicating that non-synonymous mutations were dominant in the mutant A-WW5. In general, non-synonymous mutations are mostly beneficial mutations [39,40]. By modifying 21 representative genes in the genome of Saccharomyces cerevisiae, Shen et al. found that most synonymous mutations are harmful [41]. This also indicates that these mutations may be beneficial in improving the cellulase production ability of the *A. niger* mutant A-WW5.

The gene product encoded by the GME142_g gene belongs to monooxygenase, which belongs to oxidoreductase. This enzyme requires cofactors to enhance its catalytic activity [42]. Cofactor biosynthesis is one of the important metabolic pathways of cellulose-decomposing mutant strain A-WW5. Monooxygenase is closely related to the cofactor biosynthesis pathway. Therefore, monooxygenase can improve the cellulase production ability of mutant strain A-WW5. This gene product is also related to the arachidonic acid metabolic pathway in 19 major metabolic pathways obtained with a metabolomics analysis. Studies have found that arachidonic acid can not only induce oxidative stress but also inhibit oxidative stress. Oxidative stress in turn regulates the metabolism of arachidonic acid [43].

The gene product encoded by GME8327_g belongs to NADPH-cytochrome P450 reductase (CPR), with cytochrome P450 monooxygenases (CYPs) being monooxygenases. NADPH-cytochrome P450 reductase is thus a key component of the cytochrome P450 system. CPR ensures the smooth progress of the oxidation reaction in the CYP system. In eukaryotes, cytochrome P450 reductase (CPR) is the only electron donor of all microsomal proteins, so it is an important part of the function of cytochrome P450 [44,45]. Therefore, the above two gene products play important roles in the metabolic pathway of cellulose decomposition mutant strain A-WW5 [46,47,48].

In summary, the oxidative stress caused by RONSs produced during PAW treatment also affected the mutation of *A. niger* A32 to some extent.

## 4. Conclusions

The main conclusions of this paper are as follows: PAW treatment had a significant lethal effect on *A. niger* A32; specifically, the lethality of *A. niger* A32 was as high as 98.5% after 90 min of PAW treatment and its probable mutation rate was 33.3%. The lethality and probable mutation rate of *A. niger* A32 treated with CAW were much lower than that of PAW. The microstructure of *A. niger* A32 induced by PAW treatment underwent severe crumpling and damage with the increase in treatment time, while the damage of *A. niger* A32 after CAW treatment was less severe. The effect of three long-lived particles—H_2_O_2_, NO_2_^−^, and NO_3_^−^—in PAW was not obvious. The effect of PAW on *A. niger* may be caused by other short-lived active particles, which needs further study. Plasma-activated water treatment can effectively improve the efficiency of the selection and breeding of probable mutant strains of cellulase-producing *A. niger*; 116 SNPs and 61 InDels were present in the genome of the mutant strain A-WW5 and the PAW treatment technique can provide a good reference for the treatment selection and breeding of other strains. The results of this study have guiding significance for subsequent studies of PAW in microbial mutagenesis breeding.

## Figures and Tables

**Table 1:** The concentration of H_2_O_2_, NO_2_^−^, and NO_3_^−^ in plasma-activated water (PAW) was investigated under the following conditions: a discharge voltage of 25 kV, a needle–dielectric plate distance of 3 cm, and an activation time of 180 min.

	H_2_O_2_ (mg/L)	NO_2_^−^ (mg/L)	NO_3_^−^ (mg/L)
PAW	48.76	0.046	172.36

**Table 2:** The pH, conductivity, and redox potential of plasma-activated water (PAW) and chemically activated water (CAW) with the same long-life RONS concentrations were measured under the following conditions: a discharge voltage of 25 kV, a needle–dielectric plate distance of 3 cm, and an activation time of 180 min.

	pH	Conductivity (µS/cm)	ORP (mV)
sterile water	6.39	7.15	407.27
PAW	2.42	1935	517.07
CAW	6.15	-	301.73

**Table 3:** The probable mutation rates of *A. niger* treated with plasma-activated water (PAW) and chemically activated water (CAW) with the same long-life RONS concentrations under the conditions of a discharge voltage of 25 kV, a needle–dielectric plate distance of 3 cm, and an activation time of 180 min under different treatment time.

Treatment Time (min)	30 min	60 min	90 min
PAW probable mutation rate	30.30%	30.50%	33.30%
CAW probable mutation rate	0	0	0.33%

**Table 4:** Ratio of the hyaline circle to colony diameter and enzyme activity measurements of PAW-treated strains. Different letters indicate significant differences between the sample means (*p* < 0.05).

Strain Name	HC Value	Filter Paper Enzyme Activity (U/mL)	Cellulose Endonuclease Activity (U/mL)	β-Glucosidase Enzyme Activity (U/mL)
original strain	3.33	22.48 ^h^	9.96 ^D^	26.55 ^f^
A-WW1	4.50	24.54 ^g^	11.55 ^B^	28.16 ^c^
A-WW2	5.50	25.62 ^f^	9.86 ^E^	29.83 ^b^
A-WW3	4.50	25.46 ^e^	10.12 ^C^	30.20 ^a^
A-WW4	3.50	25.98 ^d^	8.63 ^F^	22.48 ^h^
A-WW5	5.50	29.66 ^a^	13.78 ^A^	27.13 ^e^
A-WW6	4.51	28.45 ^c^	8.32 ^G^	24.57 ^g^
A-WW7	4.00	29.33 ^b^	7.62 ^H^	27.56 ^d^

**Table 5:** Ratio of the hyaline circle to colony diameter and enzyme activity measurements of CAW-treated strains. Different letters indicate significant differences between the sample means (*p* < 0.05).

Strain Name	HC Value	Filter Paper Enzyme Activity (U/mL)	Cellulose Endonuclease Activity (U/mL)	β-Glucosidase Enzyme Activity (U/mL)
original strain	3.33	22.48 ^a^	9.96 ^A^	26.55 ^a^
C-WW1	3.50	21.33 ^d^	9.54 ^D^	24.32 ^d^
C-WW2	3.51	21.44 ^c^	9.57 ^C^	24.35 ^c^
C-WW3	4.00	22.47 ^b^	9.69 ^B^	24.40 ^b^
C-WW4	3.33	20.36 ^f^	6.32 ^G^	19.37 ^h^
C-WW5	3.50	20.37 ^f^	6.59 ^F^	19.44 ^g^
C-WW6	3.33	19.88 ^g^	6.72 ^E^	20.34 ^f^
C-WW7	3.35	20.52 ^e^	6.32 ^G^	21.24 ^e^

**Table 6:** Statistics for the presence of SNPs in mutant strain A-WW5.

Sample Number	CDS Coding Region Non-Synonymous Mutations (Number)	CDS Coding Region Synonymous Mutation (Number)	Non-Coding Region (Number)	Total (Number)
A-WW5	56	13	47	116

**Table 7:** The number of base transversions and base conversions in the CDS coding region/non-coding region of the mutant strain A-WW5 was counted.

	A↔T	G↔C	A↔C	G↔T	A↔G	T↔C
CDS coding region	8	9	11	7	18	16
Non-coding region	7	9	9	6	7	9

## Data Availability

The original data presented in the study are included in the article; further inquiries can be directed to the corresponding author.

## Supplementary Materials

The following supporting information can be downloaded at https://www.mdpi.com/article/10.3390/jof10080568/s1. Figure S1. Screening step schematic diagram. Figure S2. Glucose standard curve. Table S1. Results of detection and identification of fungal species.

## References

[1] Nowinski D., Czapka T., Maliszewska I. (2024). Effect of multiple nonthermal plasma treatments of filamentous fungi on cellular phenotypic changes and phytopathogenicity. Int. J. Food Microbiol..

[2] Wang T., Wan Q.G., Xu L.H., Tian L.P., Cai M., Pu L.M. (2022). Study on the bactericidal effect and mechanism of contact glow discharge plasma on *Actinomucor elegans*. J. Radiat. Res. Technol..

[3] Xu H., Fang C., Shao C., Li L., Huang Q. (2022). Study of the synergistic effect of singlet oxygen with other plasma-generated ROS in fungi inactivation during water disinfection. Sci. Total Environ..

[4] Lunov O., Zablotskii V., Churpita O., Jäger A., Polívka L., Syková E., Terebova N., Kulikov A., Kubinová S., Dejneka A. (2016). Towards the understanding of non-thermal air plasma action: Effects on bacteria and fibroblasts. RSC Adv..

[5] Xu H., Liu C., Huang Q. (2023). Enhance the inactivation of fungi by the sequential use of cold atmospheric plasma and plasma-activated water: Synergistic effect and mechanism study. Chem. Eng. J..

[6] Yao Q., Xu H., Zhuang J., Cui D., Ma R., Jiao Z. (2023). Inhibition of Fungal Growth and Aflatoxin B_1_ Synthesis in *Aspergillus flavus* by Plasma-Activated Water. Foods.

[7] Lin C.-M., Chu Y.-C., Hsiao C.-P., Wu J.-S., Hsieh C.-W., Hou C.-Y. (2019). Optimization of plasma-activated water treatments to inactivate *Salmonella* Enteritidis (ATCC 13076) on shell eggs. Foods.

[8] Schuster E., Dunn-Coleman N., Frisvad J.C., Van Dijck P.W.M. (2002). On the safety of *Aspergillus niger*—A review). Appl. Microbiol. Biotechnol..

[9] Yu R., Liu J., Wang Y., Wang H., Zhang H. (2021). *Aspergillus niger* as a Secondary Metabolite Factory. Front. Chem..

[10] Li Y., Song Z., Zhang T., Xu W., Ding C., Chen H. (2021). Spectral characteristics of needle array-plate dielectric barrier discharge plasma and its activated water. Spectrosc..

[11] Li J., Li M., Wang L., Zhou H., Zhang X., Fu W., Zou W. (2023). Screening and identification of high cellulase-producing fungi and ultraviolet mutation selection study. Feed. Res..

[12] Xu H., Zhu Y., Du M., Wang Y., Ju S., Ma R., Jiao Z. (2021). Subcellular mechanism of microbial inactivation during water disinfection by cold atmospheric-pressure plasma. Water Res..

[13] Huang L., Guo L., Qi Y., Chen M., Niyazi G., Yang L., Zhang F., Zhang J., Yao Z., Yan J. (2021). Bactericidal effect of surface plasma under different discharge modes. Phys. Plasmas.

[14] Bai Y., Muhammad A.I., Hu Y., Koseki S., Liao X., Chen S., Ye X., Liu D., Ding T. (2020). Inactivation kinetics of *Bacillus cereus* spores by Plasma activated water (PAW). Food Res. Int..

[15] Charoux C.M.G., Patange A.D., Hinds L.M., Simpson J.C., O’Donnell C.P., Tiwari B.K. (2020). Antimicrobial effects of airborne acoustic ultrasound and plasma activated water from cold and thermal plasma systems on biofilms. Sci. Rep..

[16] Royintarat T., Choi E.H., Boonyawan D., Seesuriyachan P., Wattanutchariya W. (2020). Chemical-free and synergistic interaction of ultrasound combined with plasma-activated water (PAW) to enhance microbial inactivation in chicken meat and skin. Sci. Rep..

[17] Wang H., Zeng X.F., Feng W.H., Yu L.M., Zhai W.J., Bai W.D., Zeng L.G. (2019). Antifungal Activity and Mechanism of Limonoids from Lemon Peel against Penicillium. Food Ferment. Ind..

[18] Simoncicova J., Kalinakova B., Kovacik D., Medvecka V., Lakatos B., Krystofova S., Hoppanova L., Paluskova V., Hudecova D., Durina P. (2018). Cold plasma treatment triggers antioxidative defense system and induces changes in hyphal surface and subcellular structures of *Aspergillus flavus*. Appl. Microbiol. Biotechnol..

[19] Xia B., Vyas H.K.N., Zhou R., Zhang T., Hong J., Rothwell J.G., Rice S.A., Carter D., Ostrikov K., Cullen P.J. (2023). The importance of superoxide anion for *Escherichia coli* biofilm removal using plasma-activated water. J. Environ. Chem. Eng..

[20] Wong K.S., Chew N.S.L., Low M., Tan M.K. (2023). Plasma-Activated Water: Physicochemical Properties, Generation Techniques, and Applications. Processes.

[21] Rahman M., Hasan M.S., Islam R., Rana R., Sayem A., Sad M.A.A., Matin A., Raposo A., Zandonadi R.P., Han H. (2022). Plasma-Activated Water for Food Safety and Quality: A Review of Recent Developments. Int. J. Environ. Res. Public Health.

[22] Cheng H., Luo J., Song K., Zhao F., Liu D., Nie L., Lu X. (2022). On the dose of plasma medicine: Plasma-activated medium (PAM) and its effect on cell viability. Phys. Plasmas.

[23] Hu X., Zhang Y., Wu R.A., Liao X., Liu D., Cullen P.J., Zhou R.-W., Ding T. (2022). Diagnostic analysis of reactive species in plasma-activated water (PAW): Current advances and outlooks. J. Phys. D Appl. Phys..

[24] Guo L., Xu R.B., Gou L., Liu Z.C., Zhao Y.M., Liu D.X., Zhang L., Chen H., Kong M.G. (2018). Mechanism of Virus Inactivation by Cold Atmospheric-Pressure Plasma and Plasma-Activated Water. Appl. Environ. Microbiol..

[25] Li Y., Nie L., Liu D., Kim S., Lu X. (2022). Plasma-activated chemical solutions and their bactericidal effects. Plasma Process. Polym..

[26] Liu K., Liu S., Ran C. (2020). The Effect of Air-Water-Plasma-Jet-Activated Water on Penicillium: The Reaction of HNO_2_ and H_2_O_2_ under Acidic Condition. Front. Phys..

[27] Du M., Xu H., Zhu Y., Ma R., Jiao Z. (2020). A comparative study of the major antimicrobial agents against the yeast cells on the tissue model by helium and air surface micro-discharge plasma. AIP Adv..

[28] Wang Y., Li B., Shang H., Ma R., Zhu Y., Yang X., Ju S., Zhao W., Sun H., Zhuang J. (2022). Effective inhibition of fungal growth, deoxynivalenol biosynthesis and pathogenicity in cereal pathogen *Fusarium* spp. by cold atmospheric plasma. Chem. Eng. J..

[29] Li H., Bai G.J., Wu J., Yang H.Q., Zou W. (2019). Ultraviolet-atmospheric and room temperature plasma combined mutagenesis of cellulase-producing fungi. Food Ferment. Ind..

[30] Jiang G., Yang Z., Wang Y., Yao M., Chen Y., Xiao W., Yuan Y. (2020). Enhanced astaxanthin production in yeast via combined mutagenesis and evolution. Biochem. Eng. J..

[31] Jin J., Jia B., Yuan Y.-J. (2022). Combining nucleotide variations and structure variations for improving astaxanthin biosynthesis. Microb. Cell Factories.

[32] Zhou K., Yu C., Liang N., Xiao W., Wang Y., Yao M., Yuan Y. (2023). Adaptive Evolution and Metabolic Engineering Boost Lycopene Production in *Saccharomyces cerevisiae* via Enhanced Precursors Supply and Utilization. J. Agric. Food Chem..

[33] Xu H.H., Zhang H.B., Li H.X., Li L. (2020). The application progress of atmospheric and room temperature plasma technology in microbial mutagenesis. Adv. Biotechnol..

[34] Cai M., Wu Y., Qi H., He J., Wu Z., Xu H., Qiao M. (2021). Improving the Level of the Tyrosine Biosynthesis Pathway in *Saccharomyces cerevisiae* through *HTZ1* Knockout and Atmospheric and Room Temperature Plasma (ARTP) Mutagenesis. ACS Synth. Biol..

[35] Xu X., Niu C., Liu C., Wang J., Zheng F., Li Q. (2021). A Novel Approach to Develop Lager Yeast with Higher NADH Availability to Improve the Flavor Stability of Industrial Beer. Foods.

[36] Tian T., Wu D., Ng C.-T., Yang H., Sun J., Liu J., Lu J. (2020). A multiple-step strategy for screening *Saccharomyces cerevisiae* strains with improved acid tolerance and aroma profiles. Appl. Microbiol. Biotechnol..

[37] Guo J., Luo W., Wu X.-M., Fan J., Zhang W.-X., Suyama T. (2019). Improving RNA content of salt-tolerant *Zygosaccharomyces rouxii* by atmospheric and room temperature plasma (ARTP) mutagenesis and its application in soy sauce brewing. World J. Microbiol. Biotechnol..

[38] Xu Z., Wei J., Shen J., Liu Y., Ma R., Zhang Z., Qian S., Ma J., Lan Y., Zhang H. (2015). Genetic effects of an air discharge plasma on *Staphylococcus aureus* at the gene transcription level. Appl. Phys. Lett..

[39] Woods R., Schneider D., Winkworth C.L., Riley M.A., Lenski R.E. (2006). Tests of parallel molecular evolution in a long-term experiment with *Escherichia coli*. Proc. Natl. Acad. Sci. USA.

[40] Barrick J.E., Yu D.S., Yoon S.H., Jeong H., Oh T.K., Schneider D., Lenski R.E., Kim J.F. (2009). Genome evolution and adaptation in a long-term experiment with *Escherichia coli*. Nature.

[41] Shen X., Song S., Li C., Zhang J. (2022). Synonymous mutations in representative yeast genes are mostly strongly non-neutral. Nature.

[42] Pazmiño D.T., Winkler M., Glieder A., Fraaije M. (2010). Monooxygenases as biocatalysts: Classification, mechanistic aspects and biotechnological applications. J. Biotechnol..

[43] Tao L., Fu S.X. (2011). Research progress of arachidonic acid and oxidative stress. Chin. J. Pathophysiol..

[44] Xu Z.X., Zhu Y.G., Li S., Cheng Z. (2021). Bioinformatics Analysis of NADPH-Cytochrome P450 Reductase Genes in *Cordyceps sinensis*. Fungi Res..

[45] Maseme M.J., Pennec A., van Marwijk J., Opperman D.J., Smit M.S. (2020). CYP505E3: A Novel Self-Sufficient ω-7 In-Chain Hydroxylase). Angew. Chem.—Int. Ed..

[46] Zhang L.N., Dong L., Liao H.M. (2021). Survival strategies and related research progress of bacteria in response to excessive reactive oxygen species. Microbiol. Bull..

[47] Sies H. (2020). Oxidative Stress: Concept and Some Practical Aspects. Antioxidants.

[48] Sies H., Jones D.P. (2020). Reactive oxygen species (ROS) as pleiotropic physiological signalling agents. Nat. Rev. Mol. Cell Biol..

